# Local monitoring of photosensitizer transient states provides feedback for enhanced efficiency and targeting selectivity in photodynamic therapy

**DOI:** 10.1038/s41598-023-43625-6

**Published:** 2023-10-06

**Authors:** Elin Sandberg, Chinmaya V. Srambickal, Joachim Piguet, Haichun Liu, Jerker Widengren

**Affiliations:** Experimental Biomolecular Physics, Dept. Applied Physics, Royal Institute of Technology (KTH), Albanova Univ Center, 106 91 Stockholm, Sweden

**Keywords:** Biophysics, Analytical chemistry, Physical chemistry, Biophotonics, Imaging and sensing, Optical spectroscopy, Cancer microenvironment, Cancer therapy

## Abstract

Photodynamic therapy (PDT) fundamentally relies on local generation of PDT precursor states in added photosensitizers (PS), particularly triplet and photo-radical states. Monitoring these states in situ can provide important feedback but is difficult in practice. The states are strongly influenced by local oxygenation, pH and redox conditions, often varying significantly at PDT treatment sites. To overcome this problem, we followed local PDT precursor state populations of PS compounds, via their fluorescence intensity response to systematically varied excitation light modulation. Thereby, we could demonstrate local monitoring of PDT precursor states of methylene blue (MB) and IRdye700DX (IR700), and determined their transitions rates under different oxygenation, pH and redox conditions. By fiber-optics, using one fiber for both excitation and fluorescence detection, the triplet and photo-radical state kinetics of locally applied MB and IR700 could then be monitored in a tissue sample. Finally, potassium iodide and ascorbate were added as possible PDT adjuvants, enhancing intersystem crossing and photoreduction, respectively, and their effects on the PDT precursor states of MB and IR700 could be locally monitored. Taken together, the presented procedure overcomes current methodological limitations and can offer feedback, guiding both excitation and PDT adjuvant application, and thereby more efficient and targeted PDT treatments.

## Introduction

Photodynamic therapy (PDT) is a non- or minimally invasive therapeutic approach, where a photosensitizer (PS) upon local excitation generates cytotoxic effects. PDT is a strongly emerging treatment modality, especially for various cancer diseases, but is also faced with major challenges; (i) finite tumor suppression, (ii) poor tumor targeting, and (iii) limited therapeutic depths. To overcome these challenges, development efforts have this far focused on PS design (see^1,2^ for recent reviews). However, given the broad spectrum of different in situ conditions often encountered it is difficult to optimize PDT operation by PS design alone. Better insight into the population dynamics of PS states exerting the PDT effects, may offer additional optimization strategies, especially if the excitation and local environmental conditions can be tailored based on local feedback from the sites of treatment. However, to date such feedback has been difficult to obtain^3^.

In PDT, a PS is typically excited from a ground singlet state (S_0_) to an excited singlet state (S_1_), followed by intersystem crossing into a triplet state (T_1_). The T_1_ state can then act as a PDT precursor state, with the treatment effect strongly coupled to the T_1_ generation, by subsequent generation of cytotoxic singlet oxygen (^1^O_2_) and/or reactive oxygen species (ROS). However, the PDT effect can also be strongly coupled to the population of other photoinduced states of PS compounds, such as photo-radical states (${\dot{R}}^{+}$ and ${\dot{R}}^{-}$)^4^.

For more potent and selective PDT regimes, it is thus beneficial to promote such PDT precursor states. At the same time, they are strongly influenced by the local environment. Tumor microenvironments (TMEs) often show low O_2_ concentrations and pH, as well as enhanced levels of redox active compounds, such as glutathione and hydrogen peroxide. PDT effects of PS compounds are typically compromised under such conditions. Second, TMEs can also vary significantly, depending on e.g. stage, type and localization of the tumor, and during the course of treatments. With current PDT development, largely focusing on PS optimization by molecular design^1,2,5^, it can be difficult by PS design only, to maintain high PDT effects over the broad spectrum of different in situ conditions encountered. By design of nanoparticles (NPs), encapsulating PS compounds in a more inert environment, can offer a remedy^6^, but compared to PS molecules alone, the larger sizes of NPs often compromise biocompatibility, biodistribution and targetability.

For further adaptation, to achieve more predictable and successful PDT outcomes, one can quantify the spatial distribution of PDT excitation light onto the lesions treated and adjust fluence rates based on real-time feedback on lesion properties within specified maximum dose values (see^7,8^ for reviews). For this purpose, programs/algorithms for PDT light dosimetry have been developed, based on computational models to calculate light propagation in tissues, taking both light scattering and absorption into account^9^. Thereby, treatment-induced variations in the optical attenuation of the tissue can be obtained, providing real-time treatment feedback on how to design the excitation light for PDT^10^. Moreover, local, in situ PS concentrations can also be estimated from the fluorescence of the PS^11,12^. However, since the fluorescence brightness of the PS can vary with the local environment, absolute concentration assessments are difficult.

Beyond optimization of the PS compound itself, its mere localization/concentration, and adaptation of the excitation within the treatment region, as described above, direct in situ monitoring of the PDT precursor state populations of a PS and their rate parameters can add important orthogonal parameters for optimizing PDT effects^3^. Several spectroscopic techniques can in principle monitor such populations. Transient absorption spectroscopy (or flash photolysis, FP) has been successfully applied to monitor dark, photo-induced states of fluorophores via their absorption by a separate probing beam, following an excitation pulse^13,14^. However, overlap of absorption spectra can complicate analyses, the technique is relatively technically complicated, lacks the sensitivity for measurements at low (< µM) concentrations, and is thus mainly restricted to cuvette experiments. Thus, although suggested as a means to extract PDT precursor state information in situ^15^, FP is difficult to use for this purpose. Following emission directly (phosphorescence) or indirectly (delayed fluorescence) from T_1_ offers an additional alternative^16^, as does phosphorescence from ^1^O_2_^17^. However, compared to fluorescence, these emissions are weak and further compromised by oxygen and biomolecular quenching making them difficult to follow under in situ conditions^3^. If detectable, a PS phosphorescence signal is also not so directly coupled to PDT precursor state populations but depends on local PS concentration and quenching effects. Likewise, PS photobleaching has been suggested as a simple, cost-effective approach to monitor PDT effects^17^, but is an indirect determination, where locally diminished fluorescence can also have other origins. Moreover, once the PS is photobleached there is a limited use in adapting the excitaiton light onto the PS to optimize PDT effects.

Fluorescence Correlation Spectroscopy (FCS) offers a direct, sensitive means to follow population dynamics of dark transient states of fluorophores, via the fluorescence intensity fluctuations they generate^18^. However, since FCS relies on single-molecule detection conditions and high time resolution, only fluorophores with high brightness can be studied, and in samples where the background level is low, which limits the application range for biological sample studies.

**Figure 1:**
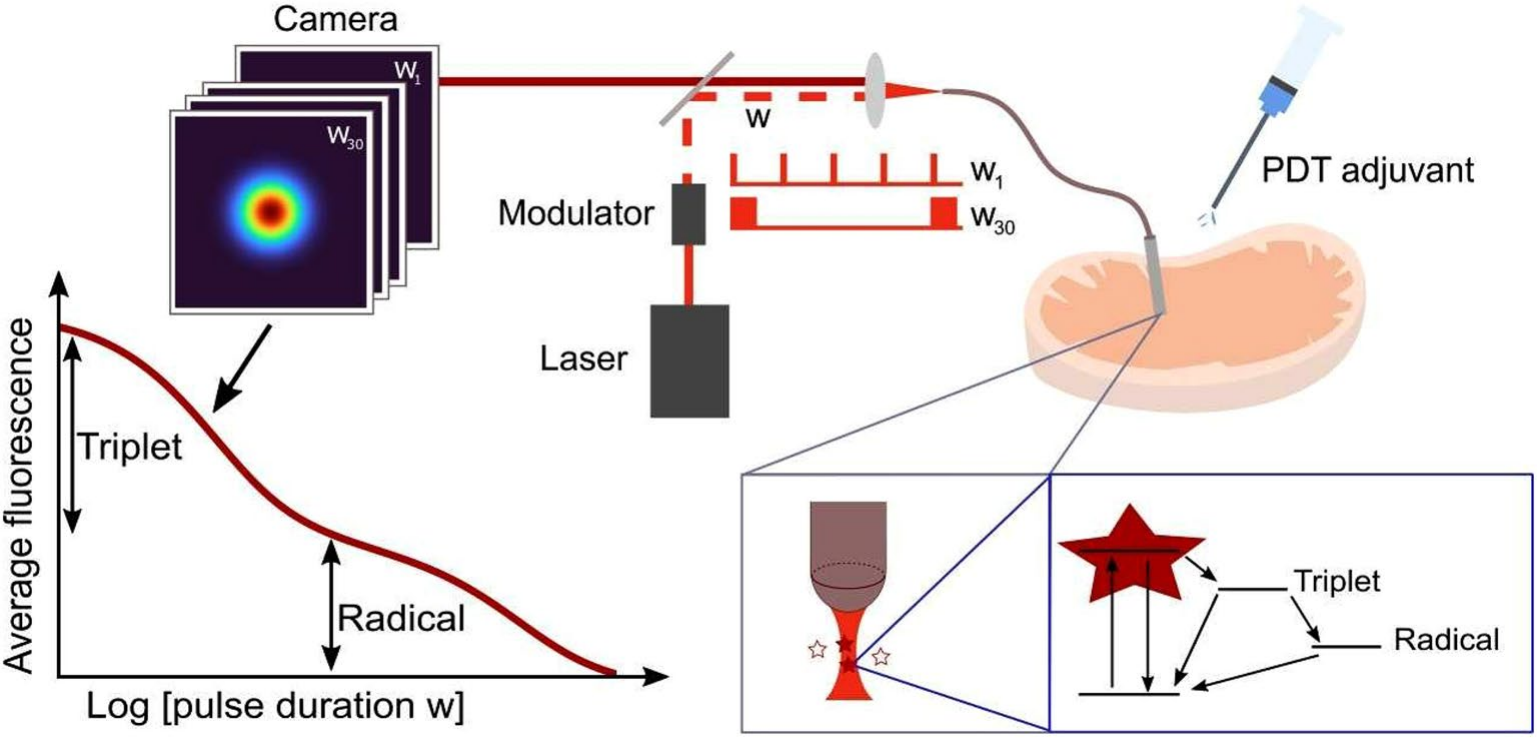
Schematic of fiber-based TRAST monitoring in tissue, how PDT precursor state formation in PS compounds are formed, and how this formation is influenced by the excitation light applied (via the same optical fiber) and by addition of different adjuvant compounds.

In this work, we propose transient state (TRAST) monitoring^19–23^, monitoring the response in a time-averaged fluorescence signal, $<F>$, to a time-modulated excitation light source, as a direct, calibration-free approach to monitor PDT precursor state populations of PS compounds in situ, at treatment locations. For demonstration, we investigated the PDT precursor state (T_1_ and ${\dot{R}}^{-}$) kinetics of two PDT agents, Methylene Blue (MB) and the silicon phthalocyanine dye IRdye700DX (IR700). MB is widely used clinically, both for fluorescence-based intraoperative imaging^24^ and as a PDT agent, active via its T_1_ state^25^. IR700 has recently emerged as an agent in photoimmunotherapy (PIT), coupled to a monoclonal antibody targeting antigens expressed on cancer cells^5^. Upon irradiation, IR700 can generate toxic ROS levels via its T_1_ state, but can also generate cytotoxic effects via an uncaging mechanism, triggered by photoreduction into an ${\dot{R}}^{-}$ state^4^. By TRAST, we established photophysical models for MB and IR700, determined rate parameters involved, and studied how these models and parameters were influenced under different environmental conditions relevant for TMEs, particularly oxygenation, pH and redox conditions. We directly show how the PDT precursor state (T_1_ and ${\dot{R}}^{-}$) populations can strongly depend on these conditions. The importance of studying excited state dynamics of PS compounds in solvents closely mimicking real physiological conditions has been highlighted^26^, and we show in this work that these populations can be monitored in a widely applicable manner in such solvents. We also introduce fiber-optically coupled TRAST measurements, with the excitation light and the generated fluorescence passing through the same fiber, and then demonstrate TRAST measurements of PDT compounds locally in tissues (schematically outlined in Fig. 1). Finally, we considered local application of various adjuvants together with a PS, affecting the PDT precursor state formation in the PS, to enhance PDT effects. Since the effects of such adjuvants can vary locally, and since they have proven difficult to monitor in situ, local application of PDT adjuvant compounds has not yet become a viable strategy to enhance PDT effects. With TRAST monitoring however, offering local information of PDT precursor state transitions, the effects of a local supply of an adjuvant can be better monitored, and thereby adapted to enhance PDT effects within a planned treatment region. This can offer flexible adjustment of the PDT to a spectrum of different TMEs that may prevail, and more generally a useful flexibility in the practice of PDT, which can be difficult to cover by PS molecular designs or other PS adaptations alone.

## Results and discussion

### Photodynamic characterization of methylene blue (MB) in solution by TRAST

**Figure 2:**
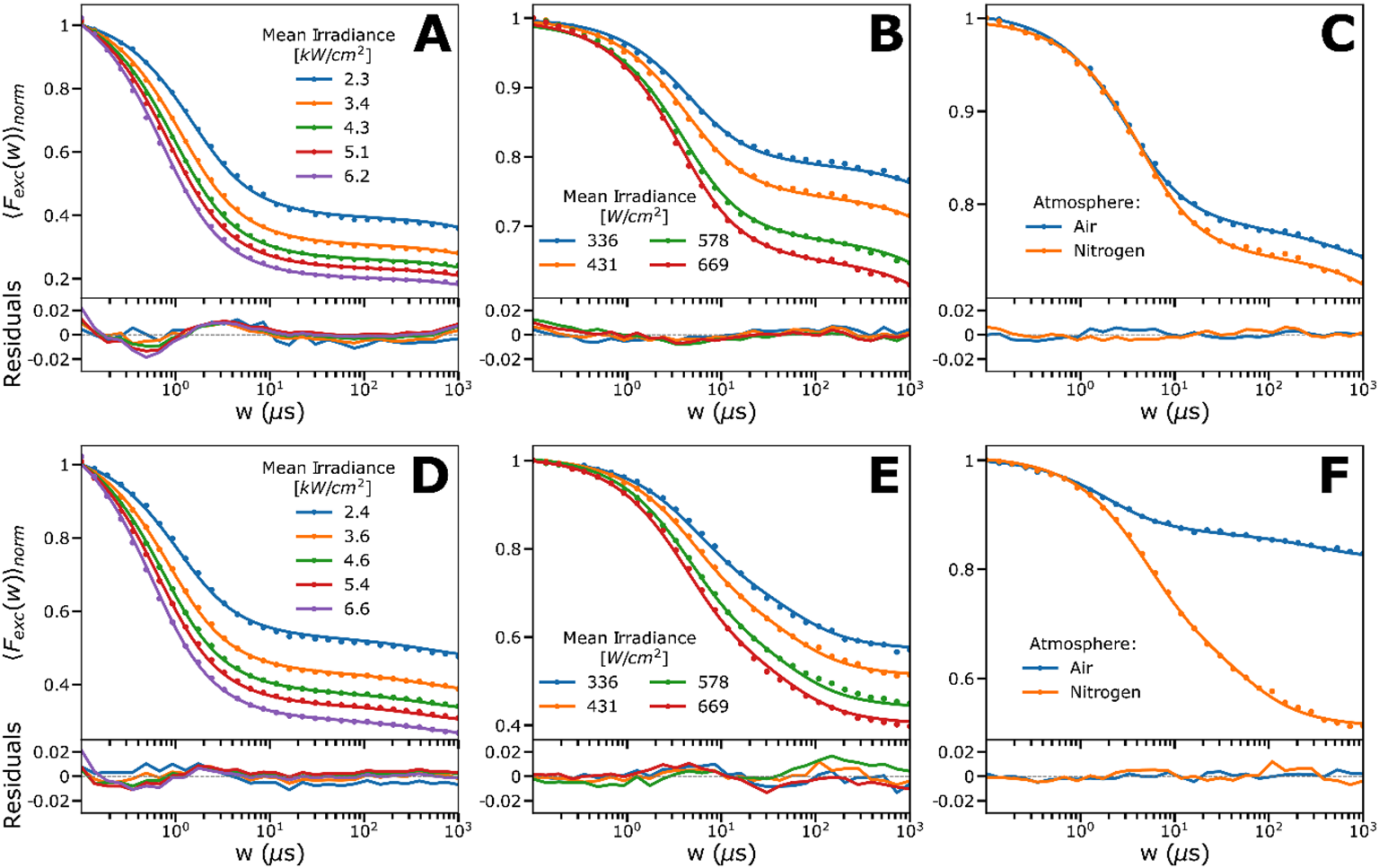
Experimental TRAST curves of MB (dissolved in 12 mM PBS). Data is represented by dots and the lines are the fitted curves, residuals below. Further details together with fitted rates are given in main text and Table 1. (**A**) Mean irradiance (*I*_*exc*_) dependence of MB at pH 3.5 (400 µM). (**B**) *I*_*exc*_ dependence of MB (8 µM) at pH 3.5 under deoxygenation. (**C**) MB (8 µM) in air-saturated solution versus nitrogen-saturated solution of pH 3.5, measured at *I*_*exc*_ = 431 W/cm^2^. Nitrogen-saturated solution shows a somewhat slower triplet decay rate than in air-saturated solution. (**D**) *I*_*exc*_ dependence of MB (400 µM) at pH 9.3. (**E**) *I*_*exc*_ dependence of MB (8 µM) at pH 9.3 under deoxygenation with nitrogen. (**F**) MB (8 µM) in air-saturated solution versus nitrogen-saturated solution of pH 9.3, measured at 431 W/cm^2^. Deoxygenation shows a highly reducing effect on the triplet decay rate compared to in air-saturated solution.

The photodynamics of MB in aqueous solutions were analyzed by widefield TRAST measurements. In TRAST, the population kinetics of dark T_1_, ${\dot{R}}^{+}$ and ${\dot{R}}^{-}$ states of fluorophores are monitored via the time-averaged fluorescence intensity, $<F>$, and from how $<F>$ changes due to population of dark states in the fluorophores upon systematic variation of the excitation modulation. By varying the excitation modulation over the same time range as the transitions of the T_1_, ${\dot{R}}^{+}$ and ${\dot{R}}^{-}$ states of the fluorophores, significant contrasts in the populations of these states (and in $<F>$) can be obtained, which is used to determine their kinetics. Typically, TRAST analyses how $<F>$ depends on the pulse duration, *w*, of rectangular excitation pulse trains in so-called TRAST curves. In such curves, with $<F>$ normalized to 1 for $w\to 0$, the decay amplitudes and times of ${<F(w)>}_{norm}$ then reflect the build-up and relaxation times of dark state populations after onset of excitation. The procedures are further described in “Materials and methods”. In contrast to FCS measurements, in which it was not possible to detect dark state relaxation in the MB samples (data not shown), clear dark state relaxations could be observed in the measured TRAST curves (Fig. 2A–F). Previous photophysical studies of MB using FP have shown that while no proton exchange of the singlet state of MB can be expected at physiological pH ranges, with $p{K}_{A}({S}_{0})\approx 0.0$, the situation is different for the triplet state, with a $p{K}_{A}({T}_{1})$ of ~ 7.2^25,27–30^. To study the effects of the protonation state of T_1_ on the dark state transitions, TRAST measurements were first performed at a pH much lower (pH = 3.5) and higher (pH = 9.3) than $p{K}_{A}({T}_{1})$. At both pH conditions, TRAST curves were recorded at different excitation intensities, ${I }_{exc}$, and under both air-saturated and deoxygenated conditions.

At low pH (Fig. 2A,B), the recorded TRAST curves showed a prominent dark state relaxation amplitude (60–80%) in the µs time range, along with a minor decay in the ms time range. The decay time of the first process decreased with higher ${I }_{exc}$ (Fig. 2A). Upon de-oxygenation (Fig. 2B), only a small increase in the first dark state relaxation amplitude was observed at corresponding ${I }_{exc}$ applied (Fig. 2C). At high pH (Fig. 2D–F), MB showed slightly lower dark state amplitudes and slower decay times than at low pH, still with increasing amplitudes and shorter decay times with higher ${I }_{exc}$ (Fig. 2D). TRAST curves recorded in deoxygenated solutions showed higher dark state amplitudes than corresponding TRAST curves at low pH, and with longer decay times than under air-saturated conditions (Fig. 2E). Upon deoxygenation, we observed a more prominent difference in the relaxation amplitude, compared to at low pH (Fig. 2F versus Fig. 2C). The ${I }_{exc}$-dependence, and the effects of deoxygenation found for MB (Fig. 2A,B,D,E) show that wide-field TRAST measurements can monitor singlet–triplet state transitions of MB, with a high rate of intersystem crossing, and identifying singlet–triplet state transitions that are different at low and high pH. Moreover, a small decay of the TRAST curves in the ms time range is observed, which can be attributed to photo-reduction of T_1_, as reported from previous FP studies^29,31^.

### Fitting of MB TRAST data into a photophysical model

**Figure 3:**
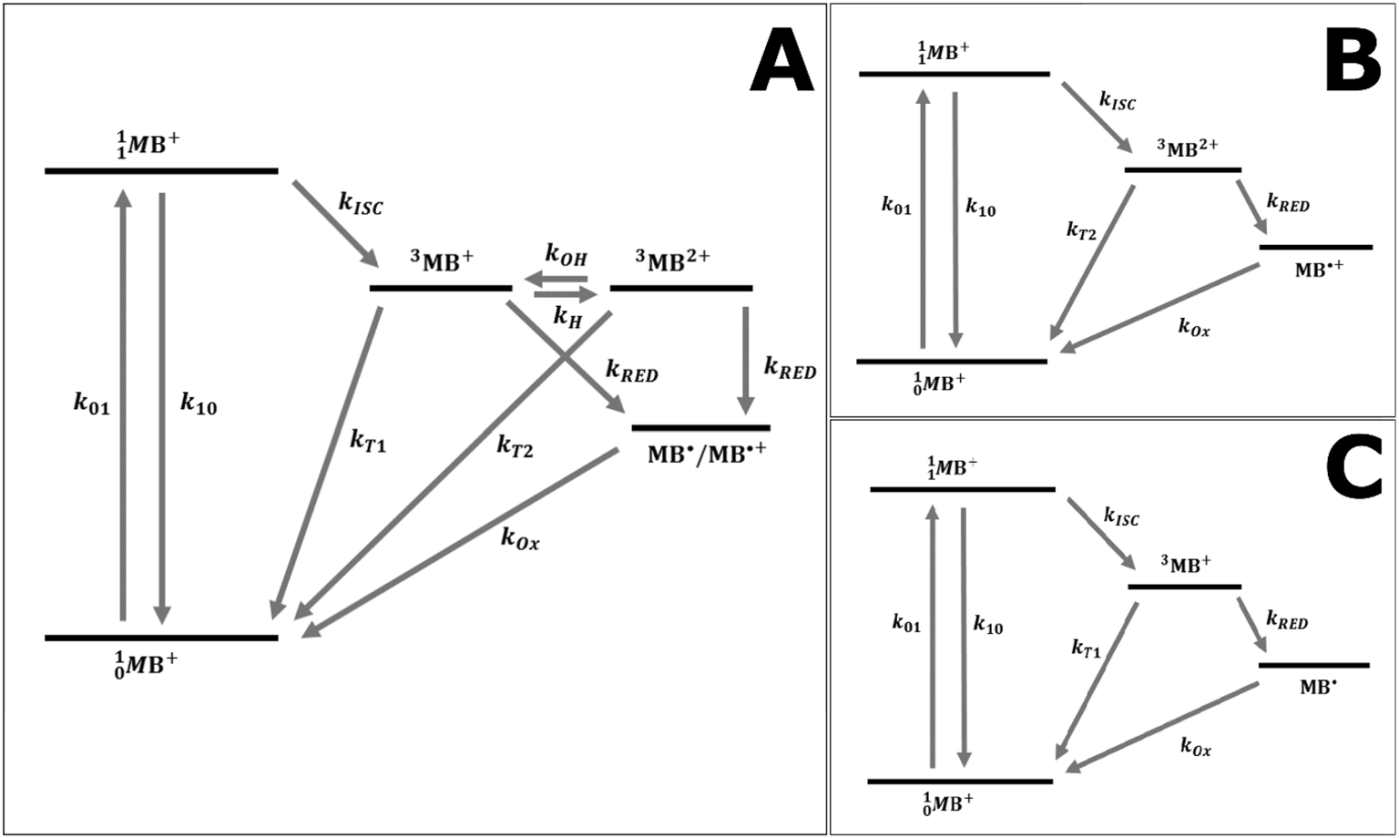
Photophysical model for MB as described in the main text. (**A**) Full model used to fit experimental buffer-titration curves obtained at pH 5, where the ratio of ${}^{3}{MB}^{+}$ and ${}^{3}{MB}^{2+}$ is sensitive to the protonation rate. (**B**) Simplified model used to fit experimental curves at low pH, where ${}^{3}{MB}^{2+}$ dominates due to fast protonation. (**C**) Simplified model used to fit experimental curves at high pH, where protonation can be neglected and ${}^{3}{MB}^{+}$ dominates.

Based on the TRAST measurements, we set up a photophysical model for MB (Fig. 3A), including a ground singlet (${S}_{0}$, also denoted ${}_{0}{}^{1}{MB}^{+}$), excited singlet (${S}_{1}$, also denoted ${}_{1}{}^{1}{MB}^{+}$), a non-protonated triplet (${T}_{1}$, also denoted ${}^{3}{MB}^{+}$), a protonated triplet (${T}_{1}H$, also denoted ${}^{3}{MBH}^{2+}$), and a photo-reduced radical state ($\dot{R}$, also denoted $\dot{MB}$ or ${\dot{MBH}}^{+})$. Depending on the pH and buffer conditions, the 5-state model of Fig. 3A can be reduced to a 4-state model, as depicted in Fig. 3B and C (see also SI, section S1). For solutions in which the pH is much lower than $p{K}_{A}({T}_{1})$, and in which the buffer concentration is high, such that the triplet protonation rate ${k}_{H}\gg {k}_{T1}, {k}_{RED1} \mathrm{and} {k}_{OH}$, the ${T}_{1}$ state will be directly protonated into ${T}_{1}H$, and the simplified model of Fig. 3B, without the ${T}_{1}$ state, can be applied. On the other end, for solutions in which the pH is much higher than $p{K}_{A}({T}_{1})$, protonation of T_1_ can be neglected. A corresponding simplified model (Fig. 3C), in this case without the ${T}_{1}H$ state, can then be used. For all models, we assume for simplicity that the photo-reduction rates from ${T}_{1}$ and ${T}_{1}H$ to $\dot{R}$ are the same (${k}_{RED}$), and also that the re-oxidation rates, from either $\dot{MB}$ or ${\dot{MBH}}^{+}$ back to ${S}_{0}$, are the same (${k}_{OX}$).

Based on these models, the population build-up of the different photo-induced, non-fluorescent states of MB can be calculated (SI, Section S1), which in turn can be used to calculate ${\langle {F}_{exc}\left(w\right)\rangle }_{norm}$ from Eqs. (2–6). In all these calculations, an excitation cross section for MB of 1.9 $\cdot 1{0}^{-16} \text{cm}^{2}$, and a fluorescence lifetime of 345 ps were used to fix the ${S}_{1}$ decay rate, ${k}_{10}$, and the excitation rate, ${k}_{01}$(see “Materials and methods”). First, calculated TRAST curves were globally fitted to the experimental TRAST curves in both Fig. 2A and D, based on the models in Fig. 3B and C, respectively. In this fit, the parameters ${k}_{ISC}, {k}_{RED} \mathrm\,{and}\, {k}_{OX}$ were globally fitted to all curves, while ${k}_{T1}$ and ${k}_{T2}$ were globally fitted to the curves at high pH (Fig. 2D) and low pH (Fig. 2A), respectively. The fitted curves could well reproduce the experimental data (Fig. 2A and D), and with the following fitted parameter values: ${k}_{ISC}$ = 1150 µs^−1^, ${k}_{T1}$ = 0.83 µs^−1^, ${k}_{T2}$ = 0.47 µs^−1^, ${k}_{RED}$ = 0.001 µs^−1^ and ${k}_{OX}$ = 0.005 µs^−1^. Next, we analyzed corresponding TRAST curves, measured at the same low and high pH, but under deoxygenated conditions (Fig. 2B and E). Global fitting was performed in the same way as for the TRAST curves recorded under air-saturated conditions, with ${k}_{RED} \mathrm\,{and}\, {k}_{OX}$ globally fitted, ${k}_{T1}$ and ${k}_{T2}$ globally fitted to the curves at high pH (Fig. 2E) and low pH (Fig. 2B), respectively, but with ${k}_{ISC}$ now fixed to 1150 µs^−1^ for all curves (as fitted above). The experimental curves could be well reproduced in the fitting, with ${k}_{T1}$ = 0.17 µs^−1^, ${k}_{T2}$ = 0.33 µs^−1^, ${k}_{RED}$ = 0.0007 µs^−1^ and ${k}_{OX}$ = 0.013 µs^−1^. Overall, the determined rates are well in line with corresponding studies on organic fluorophores, with singlet–triplet transitions in the µs time range, several orders of magnitude faster than the photo-radical state transitions^32,33^. Notably, the determined ${k}_{ISC}$ rate is several orders of magnitude higher than for most fluorophores, which agrees with previous FP studies^25,28,30,31^, and is in line with the use of MB as a photosensitizer/PDT agent. The high ${k}_{ISC}$ rate of MB, with concomitant low molecular fluorescence brightness, fast blinking dynamics and fluorescence saturation at excitation conditions for single-molecule experiments, is also the major reason why it is not feasible to characterize the MB triplet state transitions by FCS measurements^18^. With its high ${k}_{ISC}$ rate, the fluorophore brightness of MB is orders of magnitude lower than for regular fluorophores used in FCS measurements^18^, and we could consequently not retrieve any triplet state kinetics of MB from FCS, even under ideal, low background conditions (data not shown). From the TRAST measurements at low and high pH, we find that ${k}_{T1}$>${k}_{T2}$ under air-saturated conditions, but that ${k}_{T1}$<${k}_{T2}$ after deoxygenation, and that ${k}_{T1}$ and ${k}_{T2}$ are higher than the triplet state decay rates of most fluorophores following deoxygenation. This finding, and the clear difference between the kinetic properties of ${T}_{1}$ and ${T}_{1}H$, is clearly seen in Fig. 2C and F, and with only a minor effect on the ${T}_{1}H$ population upon deoxygenation. Except for somewhat higher ${k}_{T1}$ and ${k}_{T2}$ rates under deoxygenated conditions (see Table 1), which may be attributed to incomplete oxygen removal, our experiments are in agreement with previously reported FP measurements^25,28,30^, which also showed that ${k}_{T1}$<${k}_{T2}$ in absence of oxygen, and where also a several-fold higher oxygen-mediated quenching rate was found for ${T}_{1}$, compared to ${T}_{1}H$ (see Table 1).

**Table 1:** Determined rate parameter values from global non-linear least square fitting of the presented MB and IR700 photodynamic models, to TRAST PBS (12 mM) solution measurements.

Dye	Rate	Addition	Global fit	95% conf.	Ref^27^	Ref^33^	Ref^34^	Ref^42^	Unit
MB	k_ISC_	–	$1150$	$\pm 15$	2900	4000	3100		µs^−1^
MB	k_T1_	${\mathrm{OH}}^{-}$	$0.825$	$\pm 0.016$	0.65	0.33	0.7		µs^−1^
MB	k_T2_	${\mathrm{H}}^{+}$	$0.473$	$\pm 0.008$	0.16	0.33	0.29		µs^−1^
MB	k_T1_	${\mathrm{OH}}^{-},{\mathrm{ N}}_{2}$	0.171	$\pm 0.0$ 027	0.028	0.022			µs^−1^
MB	k_T2_	${\mathrm{H}}^{+}, {\mathrm{ N}}_{2}$	0.325	$\pm 0.0$ 071	0.2	0.2			µs^−1^
MB	k_RED_	–	$0.00096$	$\pm 0.00028$					µs^−1^
MB	k_OX_	–	$0.00507$	$\pm 0.002$					µs^−1^
MB	k_H_	PBS	$0.508$	$\pm 0.124$	46		20		mM^−1^ µs^−1^
IR700	k_ISC_	–	$65.1$	$\pm 1.03$				3.8	µs^−1^
IR700	k_T_	–	$0.542$	$\pm 0.00765$				0.56	µs^−1^
IR700	k_RED_	Asc^−^	$0.11$	$\pm 0.004$					mM^−1^ µs^−1^
IR700	k_OX_	Asc^−^	$0.00468$	$\pm 0.000263$					µs^−1^

The uncertainty is reflected by given confidence intervals. All curves were measured at 400 nM. Some reference results are included as a comparison, where the reported results have been recalculated to have the same unit and dimension as our reported values.

### pH-dependence of MB photodynamics

**Figure 4:**
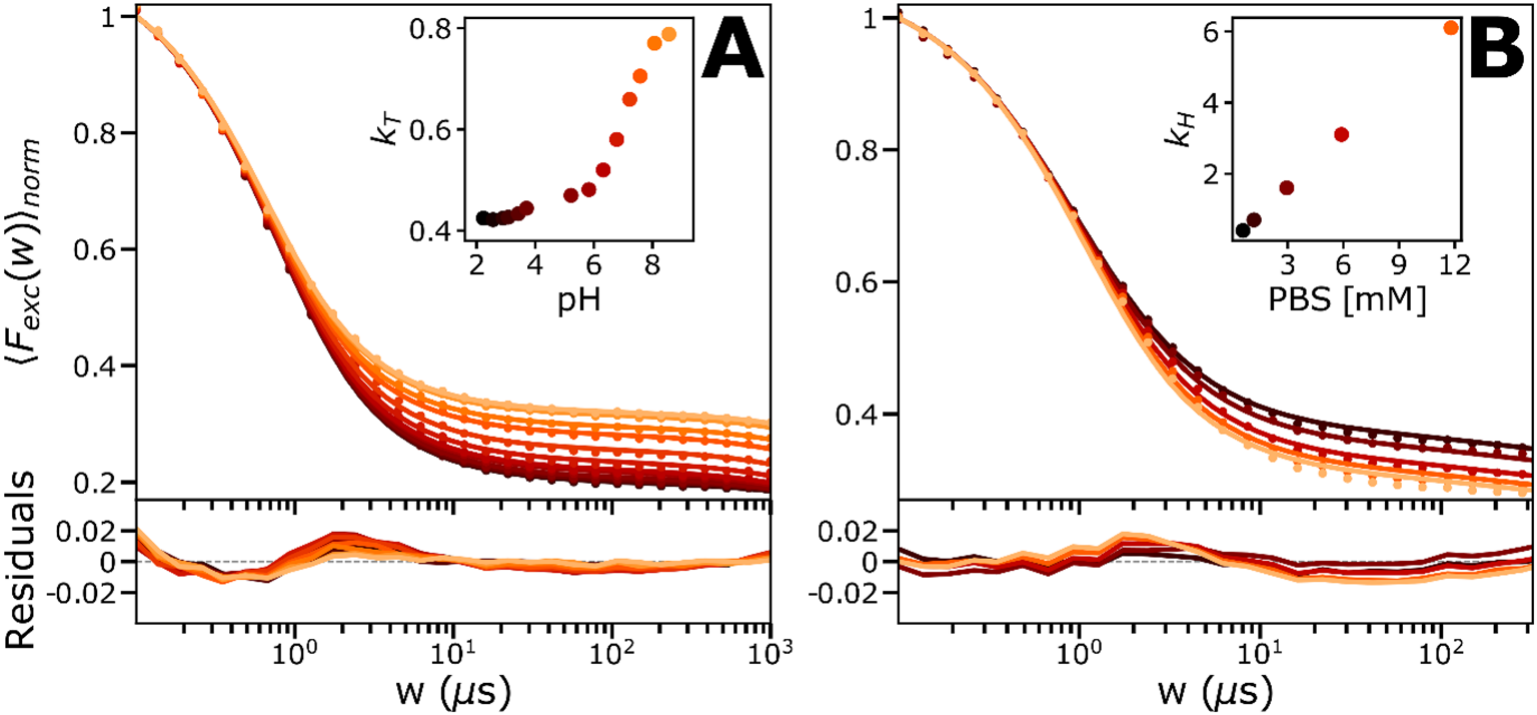
Experimental TRAST curves of MB (dissolved in air-saturated PBS). Data is represented by dots and the lines are the fitted curves, residuals below. See main text for further details. (**A**) Curves showing the pH-dependence of MB (in 12 mM PBS), fitted with a 4-state model, with all rates fixed to the globally fitted values, except the ${k}_{T}$-rate. Here the ${k}_{T}$-rate is a linear combination of ${k}_{T1}$ and ${k}_{T2}$, shown in inset with a triplet state pKa of around 7.2. (**B**) Curves showing the PBS buffer concentration-dependence of MB, fitted with the full 5-state model, with all rates fixed to previously fitted values and a linear dependence with the buffer concentration was put on ${k}_{H}$, shown as inset, where the slope and the intercept were fitted as free parameters.

Next, we studied how the different kinetics of the ${T}_{1}$ and ${T}_{1}H$ states influenced the TRAST curves, when recorded over a pH range of 2 to 9. In TRAST curves, recorded in air-saturated buffer solutions, we found that the triplet state relaxation amplitude decreased with increasing pH (Fig. 4A). In presence of buffer (PBS, 12 mM), the exchange rates ${k}_{H}$ and ${k}_{OH}$ between ${T}_{1}$ and ${T}_{1}H$ can be considered much faster than the other rates from these states. Then, a 4-state model (Fig. 3B or C) can be used, including a compound triplet state decay rate, which is a linear combination of ${k}_{T1}$ and ${k}_{T2}$, weighted by the protonated and unprotonated fractions of the triplet state MB, respectively:

1 $${k}_{T}=\frac{{T}_{1}}{{T}_{1}+{T}_{1}H}\cdot {k}_{T1}+\frac{{T}_{1}H}{{T}_{1}+{T}_{1}H}\cdot {k}_{T2}.$$

Calculated curves based on this model were fitted to the experimental curves (Fig. 4A), with ${k}_{ISC}, {k}_{RED} \mathrm\,{and}\, {k}_{OX}$ set global and fixed to the values determined from the data in Fig. 2A and D, and with ${k}_{T}$ individually fitted to each curve. With only one freely fitted parameter per fitted curve, they could well reproduce the experimental TRAST curves (Fig. 4A), with fitted ${k}_{T}$ values well in agreement with Eq. (1) (Fig. 4A, inset), and a pK_A_ for the triplet state of ~ 7.2, as reported^25,27–30^. To investigate the buffer dependence, we then recorded TRAST curves from MB at pH 5, at buffer concentrations varying between 0 and 12 mM (Fig. 4B). We then used the model of Fig. 3A to calculate curves fitted to the experimental TRAST curves. In the fitting, ${k}_{ISC}, {k}_{RED}, {k}_{OX}$, ${k}_{T1}$ and ${k}_{T2}$ were all globally fixed to their determined values from Fig. 2A and D. The triplet protonation rate, ${k}_{H}$, was globally fitted to be linearly dependent on the buffer concentration, ${k}_{H}={k}_{H}\left(0\right)+{k}_{H-PBS}\cdot \left[\mathrm{PBS}\right]$, and with ${k}_{OH}$ fixed by ${k}_{H}$ to: ${{k}_{OH}=k}_{H}\cdot 1{0}^{\left[pH-p{K}_{A}\left({T}_{1}\right)\right]}$, with $p{K}_{A}\left({T}_{1}\right)$ set to 7.2. With only two free parameters (${k}_{H}\left(0\right)\mathrm\,{ and}\, {k}_{H-PBS}$), it was still possible to well reproduce all five experimental TRAST curves (Fig. 4B, with the fitted ${k}_{H}\left(0\right)$ and ${k}_{H-PBS}$ given by the intercept and slope in the inset), with the fit yielding ${k}_{H}\left(0\right)=$ 0.1 µs^−1^ and ${k}_{H-PBS}=0.5\cdot {10}^{9}\, \text{M}^{-1} \text{s}^{-1}$.

### Effects of potassium iodide, as a possible PDT adjuvant together with MB

**Figure 5:**
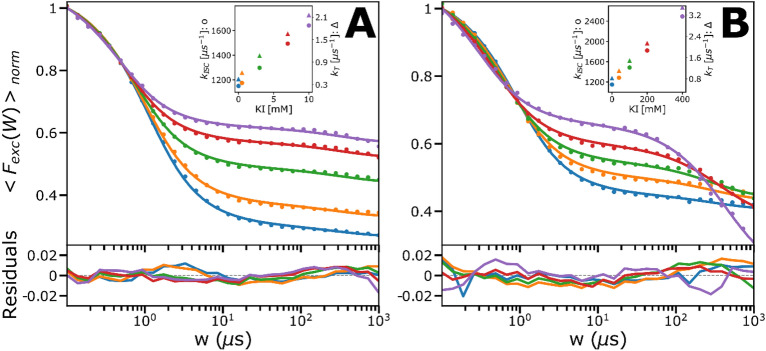
Measured (dots) and fitted (lines) TRAST curves with residuals below, of MB (dissolved in air-saturated PBS, 12 mM) at high and low pH, in the presence of KI as a potential adjuvant. The triplet states for both sets of curves are however decreasing, which can be explained by a higher relative increase in ${k}_{T1}$/${k}_{T2}$ due to a charge-transfer mediated effect, compared to the increase of ${k}_{ISC}$ due to the heavy-atom effect (shown as insets where ${k}_{ISC}$ is represented by dots and ${k}_{T}$ by triangles). KI also has antioxidizing effects, why ${k}_{RED}$ also showed a linear dependence. See main text for further details. (**A**) KI-titration (0–10 mM) series at pH 4. (**B**) KI-titration (0–400 mM) series at pH 9. The triplet state population is decreasing to a lower extent than at high pH, which potentially could be due to lower electrostatic interactions compared to at low pH, where the protonated form dominates.

Next, we studied effects on the PDT precursor states of MB (${T}_{1}$, ${T}_{1}H$ and $\dot{R}$) upon adding potassium iodide (KI). KI is a well-known fluorescence quencher, enhancing ISC rates of fluorophores by a so-called heavy-atom effect^34^, and may thus potentially serve as a PDT adjuvant. To investigate possible enhancement effects of KI on the ${T}_{1}$, ${T}_{1}H$ and $\dot{R}$ states of MB, we recorded TRAST curves from MB, at low (4) and high (9) pH, and with different KI concentrations, [KI], added (Fig. 5A and B). With increased [KI], despite an expected heavy atom ISC enhancement effect, the triplet state populations were found to decrease, for both pH conditions. This effect was stronger at low pH. For both pH conditions also a minor increase in $\dot{R}$ could be observed. Each set of TRAST curves, at low pH (Fig. 5A) and high pH (Fig. 5B), were fitted globally, using the model in Fig. 3B and C, respectively.

In the fitting, ${k}_{T1}$/${k}_{T2}$ was individually fitted to the curves, given that KI has been found to promote ${k}_{T}$ for some fluorophores, via a charge-transfer mediated effect^32^. KI has also been found to act as an anti-oxidant, why ${k}_{RED}$ together with ${k}_{ISC}$ were fitted globally, with a linear dependence on [KI] (i.e. as ${k}_{X}={k}_{X}(0)+{k}_{QX}\left(KI\right)\cdot \left[KI\right]$, with X = RED or ISC1 (low pH) or ISC2 (high pH)). The fitted curves could well reproduce the experimental TRAST curves, with the following fitted parameters: ${k}_{RED}$ = 0.001 µs^−1^, ${k}_{ISC1}$ = ${k}_{ISC2}=$ 1150 µs^−1^, ${k}_{QISC1}$ = 49.1 × 10^9^ M^−1^ s^−1^, ${k}_{QISC2}$ = 3.4 × 10^9^ M^−1^ s^−1^, and ${k}_{QRED}$ = 0.00001 × 10^9^ M^−1^ s^−1^. From the fits, it can be noted that the triplet state decay rates also depend linearly on [KI]. Moreover, ${k}_{QT1}$>>${k}_{QT2}$ (see insets of Fig. 5A and B), and also ${k}_{QISC}$ is much higher at low pH than at high pH (same insets). One possible reason is that stronger electrostatic attractions can be expected between (negatively charged) iodide ions and (protonated) MB at low pH. Upon adding KI, the enhancement of ${k}_{ISC}$ in absolute numbers is much higher than of ${k}_{T1}$/${k}_{T2}$. However, given the high intrinsic ISC rate of MB, the relative increases in ${k}_{T1}$/${k}_{T2}$ is higher than in ${k}_{ISC}$. Since the resulting triplet state populations are largely determined by the ratio between ${k}_{T1}$/${k}_{T2}$ and ${k}_{ISC}$ this explains the decreased triplet state populations of MB with increasing [KI] (see SI, section S2 for further discussion). The increase in ${k}_{RED}$ with increasing [KI], and that it is somewhat higher from ${T}_{1}H$ than from ${T}_{1}$, is consistent with KI acting as a reductant, more strongly acting on the (more positively charged) ${T}_{1}H$. In all, despite its well-known heavy atom enhancement effects on ISC rates of fluorophores, our investigations show that adding KI leads to an overall reduction of the triplet state population of MB. While this triplet reduction effect itself may speak against the use of KI as a PDT adjuvant of MB, adjuvant effects cannot be excluded. In fact, KI has been reported to potentiate antimicrobial photodynamic inactivation when added together with MB, attributed to singlet oxygen-mediated formation of iodine radicals and molecular iodines^35^. Such formation, by a concerted action of MB and KI, is not detected by our TRAST method but may be indirectly reflected in how the transient state kinetics of MB is altered by KI.

### Photodynamic characterization of IRdye700DX (IR700) in solution

**Figure 6:**
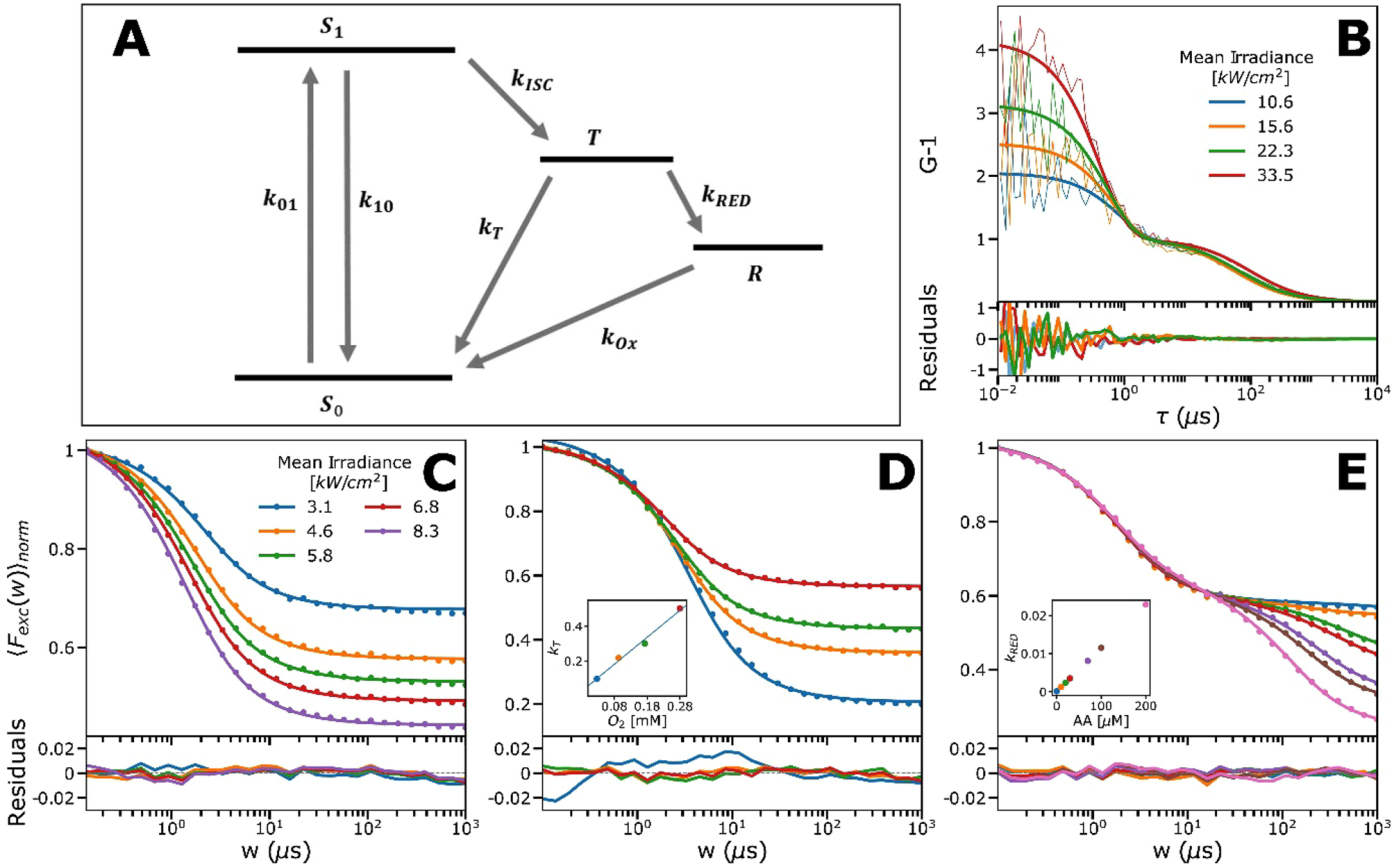
Electronic state model for IR700, together with experimental FCS and TRAST curves recorded from IR700 (dissolved in 12 mM PBS). TRAST data is represented by dots and the lines are the fitted curves with residuals below (**C**–**E**). For further details and fitted rates, see the main text and Table 1. (**A**) IR700 electronic state model, in which the singlet ground state (S_0_) is excited to the emissive singlet excited state (S_1_) from where it via ISC can go to the triplet state ${\mathrm{T}}_{1}$. From ${\mathrm{T}}_{1}$ IR700 can again return to S_0_ or form a photo-reduced, radical anion state (R). From R it can be re-oxidized back to the ground state, or the release of a phenol ligand can be triggered. (**B**) *I*_*exc*_ dependence of IR700 (~ 10 nM) measured with FCS. Fits are represented by thick solid lines and residuals to the fitted data below. The FCS-data was fitted globally by a triplet-state model. (**C**) *I*_*exc*_ dependence measured with TRAST. Curves were fitted globally to a triplet-state model, where the rates agreed with those obtained from the fitted FCS-curves. (**D**) Percentage of dissolved oxygen is varied by mixing the surrounding air with nitrogen. Inset is the ${k}_{T}$-rate versus the dissolved oxygen concentration, that shows a linear dependence. Fitted data gave the oxygen quenching rate: ${k}_{q,{O}_{2}}=1.5\cdot {10}^{9}\;\; {\mathrm{M}}^{-1}{\mathrm{s}}^{-1}$. (**E**) Titration of ascorbic acid. Here, the curves were fitted with a triplet state and a reduced state, which clearly appeared when ascorbic acid was added. ${k}_{RED}$ (inset) versus the ascorbic acid (AA) concentration, showed a linear dependence, as can be expected. All curves in Figure (**C**) and (**D**) were measured with *I*_*exc*_ = 4.5 kW/cm^2^.

Similar to MB, we performed widefield TRAST and also FCS solution experiments on the PDT precursor state dynamics of the NIR silicon phtalocyanine IR700, used as a PIT photosensitizer^5^. Apart from a type II PDT mechanism via ISC to its lowest triplet state, ${T}_{1}$_,_ a key PDT precursor state of IR700 is the radical anion state, ${\dot{R}}^{-}$. ${\dot{R}}^{-}$ is formed from ${T}_{1}$^4,36,37^, and can trigger release of a phenol ligand from the axially unsymmetrical IR700, which in turn can generate membrane damage. As for the MB experiments above, we could thus use a 4-state model to analyze the kinetics of the ${T}_{1}$ and ${\dot{R}}^{-}$ formation in IR700, with ${S}_{0}$ and ${S}_{1}$ as the other two states in the model (Fig. 6A). First, we performed FCS experiments on IR700 in air-saturated PBS solutions. In contrast to MB, prominent dark state relaxations were clearly observed in the FCS experiments (Fig. 6B), with relative amplitudes exceeding 70% at higher ${I }_{exc}$ applied (> 30 kW/cm^2^). The ${I }_{exc}$-dependence of these relaxations, with increasing amplitudes and shorter relaxation times with higher ${I }_{exc}$, suggested that they are due to singlet–triplet transitions^18^. The second decay in the FCS curves, due to translational diffusion of IR700 into and out of the detection volume (${\tau }_{D}$~ 70 µs), had longer decay times at higher ${I }_{exc}$, indicating an enlarged detection volume caused by fluorescence saturation broadening^18^. No relaxation due to ${\dot{R}}^{-}$ formation was observed in the FCS curves, indicating that this formation takes place at much longer time scales than the singlet–triplet transitions, and beyond the transit times of IR700 through the detection volume (> ${\tau }_{D}$).

Hence, the FCS curves were fitted to a 3-state model, omitting the ${\dot{R}}^{-}$ state in Fig. 6A, and using SI Section 4, Eqs. S16–S18, with one diffusion term and a triplet state decay term. In these analyses, as well as in the analyses of the TRAST measurements of IR700 described below, an excitation cross section for IR700 of 0.76 $\cdot 1{0}^{-16} \text{cm}^{2}$, and a fluorescence lifetime of 4 ns were used to fix the ${S}_{1}$ decay rate, ${k}_{10}$, and the excitation rate, ${k}_{01}$ (see “Materials and methods”). As described by Eq. S3, non-uniform ${I }_{exc}$ and fluorescence saturation effects were accounted for in the fitting, in which ${\tau }_{D}$ was fitted individually to each FCS curve, and ${k}_{T}$ and ${k}_{ISC}$ were fitted globally. The fitting yielded an ISC rate of ${k}_{ISC}$ = 65 µs^−1^, which is in the same range as reported for other Si-phtalocyanines^38,39^, but one order of magnitude higher than in recently reported FCS experiments^40^. This difference likely illustrates the difficulty in FCS experiments to determine fluorophores with large ISC rates (like IR700), and in which case corrections for fluorescence saturation and non-uniform ${I }_{exc}$ in the detection volume becomes critical^18^. Thus, by proper corrections and when measured under SMD conditions, the triplet state kinetics of IR700 can still be determined by FCS. For fluorophores with yet higher ISC rates, however, this becomes increasingly difficult (as for MB, showing more than an order of magnitude higher ISC rates than IR700). The triplet decay rate of IR700 was determined to ${k}_{T}$ = 0.54 µs^−1^, which is well in agreement with a triplet state decay promoted by molecular oxygen, and similar to other organic fluorophores^18^.

We then performed widefield TRAST measurements on IR700, as for MB (described in “Materials and methods”), which confirmed the FCS experimental data, with TRAST curves showing a prominent decay, with increased amplitude and shorter decay time with higher ${I }_{exc}$ (Fig. 6C). No obvious, additional decay was observed in the TRAST curves, and thus the same model as for the FCS curve fitting (with ${S}_{0}$, ${S}_{1}$ and ${T}_{1}$) could be used. Calculated TRAST curves according to this model could be well fitted to the experimental TRAST curves (Fig. 6C) with ${k}_{ISC}$ = 65 µs^−1^ and ${k}_{T}$ = 0.54 µs^−1^, very well in agreement with the FCS analyses. Similarly, in TRAST curves recorded from IR700 under lowered oxygen concentrations also no ${\dot{R}}^{-}$ formation was evident (Fig. 6D). We could thus use the same 3-state model also in the fitting of these curves. This fit, with ${k}_{ISC}$ fixed to 65 µs^−1^, as determined above, and ${k}_{T}$ freely fitted, could well reproduce the experimental TRAST curves, yielding an expected linear dependence of ${k}_{T}$ on the oxygen concentration, with ${k}_{T}={k}_{T}+{k}_{QT}\cdot [{O}_{2}]$ (inset Fig. 6D). Next, and given the relevance of ${\dot{R}}^{-}$ formation for the PDT effect of IR700, we studied possible PDT adjuvant effects upon adding a reductant, sodium ascorbate (AA^−^). Indeed, then a clear, second decay in the sub-ms time range was observed in the TRAST curves, showing increased amplitudes with higher AA^−^ concentrations (Fig. 6E), consistent with enhanced ${\dot{R}}^{-}$ formation. To accommodate this effect in the curve fitting, we could then use the 4-state model of Fig. 6A, including ${\dot{R}}^{-}$. The fit, with ${k}_{ISC}$ fixed to 65 µs^−1^, ${k}_{OX}$ and ${k}_{RED}$ globally fitted, where ${k}_{RED}$ was assumed to depend linearly on the AA^−^ concentration, and ${k}_{T}$ individually fitted to each TRAST curve, could well reproduce the experimental TRAST curves, with ${k}_{RED}$ = 0.004 mM^−1^ µs^−1^, ${k}_{OX}$ = 0.0047 µs^−1^, and ${k}_{T}$ = 0.58 ± 0.04 µs^−1^ (Fig. 6E, with ${k}_{RED}$ plotted versus AA^−^ in inset). In contrast to KI and MB, the TRAST data in Fig. 6E thus suggest that AA^−^ can be a useful PDT adjuvant of IR700, promoting its PDT precursor state ${\dot{R}}^{-}$.

### Fiber-coupled TRAST measurements in solutions and tissue

**Figure 7:**
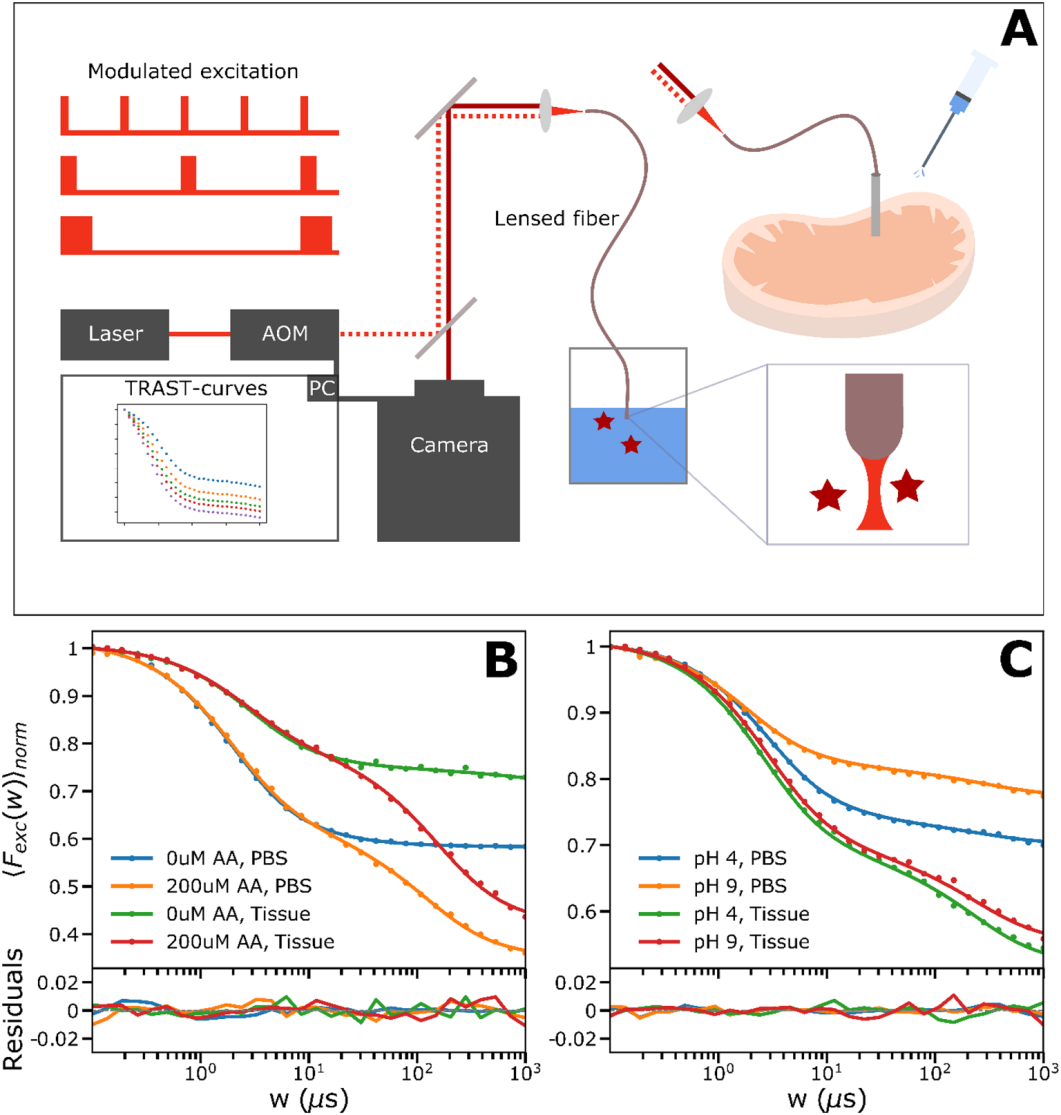
(**A**) Experimental setup used for the fiber based TRAST measurements. Instead of focusing the modulated beam onto the back-focal-plane of the objective, as for the widefield-measurements, it is sent through a multimode fiber that is focusing the excitation beam and collecting the emission that is imaged onto the camera. The fiber is either kept in solution with the dissolved dye or inside a piece of tissue through a cannula. A more detailed description can be found in “Materials and methods”. (**B**) Fiber-based TRAST curves of 2 µM IR700 obtained in tissue and PBS with and without ascorbic acid. Data is represented by dots and fits by solid lines. The triplet state population is reduced in tissue and ascorbic acid clearly contributes to the generation of the photo-reduced state also in tissue. (**C**) Fiber-based TRAST curves of 8 µM MB diluted in different pH, acquired in tissue and PBS. Data is represented by dots and fits by solid lines. The triplet state population as well as photoreduction is enhanced in tissue compared to in solution and the pH has a smaller effect in tissue than that observed in solution.

The widefield TRAST measurements, as described above, show that the PDT precursor state (T_1_ and ${\dot{R}}^{-}$) transitions of MB and IR700 can be quantitatively and reliably monitored. We note that the experimental setup and procedure used is much simpler than for FP. Moreover, compared to FCS measurements, the TRAST analyses do not fall short on compounds with high ISC (a property of most PDT agents), do not require single-molecule detection conditions, or a high time resolution. Based on these prerequisites, we modified our widefield TRAST setup into an optical multimode fiber-coupled system (fiber-TRAST), as a possible strategy to monitor PDT precursor states of PS compounds locally, at sites of treatment (Fig. 7A, further described in “Materials and methods”). Thereby, we also wanted to indicate how locally applied TRAST monitoring can provide feedback guiding the excitation, or addition of PDT precursor state adjuvants applied at the same site, to locally enhance the population of the PDT precursor states and possibly the PDT treatment effects.

First, we performed fiber-TRAST measurements, placing the fiber-end into aqueous solutions with MB and IR700, and compared the recorded curves with curves recorded from the corresponding samples using widefield TRAST. Thereby, a scaling factor for the focal beam diameter in the fiber-TRAST experiments was determined, accounting for the different geometries of the excitation and collected fluorescence light in the fiber-TRAST measurements (see “Materials and methods”). TRAST curves recorded by fiber-TRAST from MB and IR700 in solution at different ${I }_{exc}$ were found to well reproduce corresponding curves recorded by widefield TRAST (SI Section S3, Fig. S1) and yielded very similar fitted rate parameters (Table 2). Effects of pH (for MB) and AA^−^ (for IR700) could also be well reproduced (Fig. 7B and C). Next, we performed fiber-TRAST measurements in a fresh loin of pork. Prior to the measurements, a PBS-solution with 8 µM MB or 2 µM IR700 was injected into the sample through a cannula. The optical fiber was then inserted through the same cannula. In the IR700 measurements, somewhat lower triplet state populations were found in this sample, compared to corresponding PBS measurements (Fig. 7B).

**Table 2:** Determined rate parameter values for the fiber-TRAST measurements of the presented MB and IR700 photodynamic models obtained for MB and IR700 in PBS (12 mM) solution.

Rate	Addition	Global fit	95% conf.	Unit	Dye
*k*_*ISC*_	*–*	$1150$	$\pm 25$	µs^−1^	MB 4 µM
*k*_*T1*_	*–*	$0.743$	$\pm 0.02$	µs^−1^	MB 4 µM
*k*_*T2*_	*–*	$0.488$	$\pm 0.0115$	µs^−1^	MB 4 µM
*k*_*RED*_	*–*	$0.000905$	$\pm 0.000188$	µs^−1^	MB 4 µM
*k*_*OX*_	*–*	$0.00321$	$\pm 0.00107$	µs^−1^	MB 4 µM
*k*_*ISC*_	*–*	$88$	$\pm 2.53$	µs^−1^	IR700 1 µM
*k*_*T*_	*–*	$0.539$	$\pm 0.014$	µs^−1^	IR700 1 µM

The uncertainty is reflected by given confidence intervals.

Tissue measurements of MB gave a slightly higher intersystem crossing-rate of 1550 instead of 1150 as was obtained in solution. The triplet decay rate when adding MB solution of different pH to the tissue only changed from 0.47 (pH 9) to 0.42 (pH 4). Tissue measurements of IR700 gave around 50% lower intersystem crossing-rate (45 µs^−1^) than in solution, while the triplet decay rate remained similar to that obtained in solution. Reduction and oxidation rates were in a similar range. (${\mathrm{k}}_{\mathrm{RED}}$ = 0.0139 µs^−1^ in PBS, ${\mathrm{k}}_{\mathrm{RED}}$ = 0.0126 µs^−1^ in tissue, ${\mathrm{k}}_{\mathrm{OX}}$ = 0.00793 µs^−1^ in PBS, ${\mathrm{k}}_{\mathrm{OX}}$ = 0.00923 µs^−1^ in tissue).

A significant effect was seen upon adding AA^−^ (200 µM), showing how differences in local (redox) conditions, and local changes in PDT precursor state populations (${\dot{R}}^{-}$) can be monitored and generated in this sample. Moreover, local rate parameters for the ${T}_{1}$ and ${\dot{R}}^{-}$ state transitions could be determined (caption, Table 2) by fitting, based on the 4-state model in SI Section 1, Fig. 6A. For MB, larger triplet state populations, as well as more pronounced photoreduction were observed in the tissue sample, compared to corresponding PBS measurements (Fig. 6D). Further, in contrast to solution measurements, no major difference could be detected in the TRAST curves upon injecting PBS solutions with a low and high pH (4 and 9). MB is prone to adhere to and pass over membranes into cells and organelles^30^. The recorded TRAST curves in the tissue sample may thus reflect that MB is more integrated into the sample, and experiences a different environment than in solution, with lower oxygen quenching of ${T}_{1}$, a higher anti-oxidative effect promoting ${\dot{R}}^{-}$, and with an inherent buffering capacity in the tissue, which neutralizes the added PBS buffers.

## Conclusions

We show in this study that photodynamics of PDT agents with high ISC rates can be quantitatively analyzed by TRAST monitoring, in solutions and in tissue. Such monitoring is difficult, if possible at all, with other methods, such as FP and FCS. This opens the possibility to monitor buildup of PDT precursor states in PS compounds used for PDT, at locations where the PDT is to take place, and during the course of a treatment. Several environmental factors, such as oxygenation, redox conditions and pH tend to vary strongly both in time and space at and around treatment locations for PDT, and we observe in this work that these conditions can also largely influence central PDT precursor state transitions of PS compounds. Adapting the excitation modulation, based on feedback about the PDT precursor state populations from TRAST monitoring, may thus help to enhance PDT locally at the intended treatment location, while at the same time reducing the PDT effects in surrounding regions (where the environmental conditions may be found to be different, or where else the excitation can be applied differently to reduce the PDT effects). Finally, we also investigated effects of two potential PDT adjuvants, KI and AA^−^, where at least addition of AA^−^, in solutions as well as in tissue, was found to enhance the buildup of a PDT precursor state (${\dot{R}}^{-}$ in IR700). Yet, the investigation of KI shows that it can be difficult to predict adjuvant effects of an added compound, particularly in the often complex environment where PDT is to take place, and even in well-defined solutions studies. With the fiber-TRAST approach presented in this work, PDT enhancement effects of possible adjuvants can be monitored locally, thereby providing feedback both for the local supply of adjuvants and for excitation conditions for optimizing the PDT effects. In a future scenario, PDT treatments with optical fibers directed to the site of treatment, for TRAST monitoring, for delivery of excitation light, and also for delivery of adjuvants (via the channels within hollow optical fibers, or by parallel channels accompanying the optical fibers) can thus be a viable approach to enhance PDT treatment effects and selectivity.

## Materials and methods

### Sample preparation

Powder stocks of Methylene Blue (MB, Sigma M9140) and IRdye700DX (IRDYE700DX; LI-COR 929-70010) were prepared as stock solutions of 8 mM MB and 500 µM IRdye700DX using 12 mM phosphate buffer (PBS, pH 7.2) and stored at − 20 °C. Fresh samples were prepared daily before measurements with dilution using the same phosphate buffer adjusted to the desired pH. TRAST solution measurements were performed at a final concentration of 400 nM MB or IRdye700DX if not stated otherwise, put in an 8-well slide (approx. 500 µL) to prevent evaporation. Solutions of potassium iodide (KI, Sigma T0254), and sodium L-ascorbate (AA^−^, Sigma 11140) were prepared directly before experiments, using the above phosphate buffer.

Optical fiber coupled TRAST measurements (fiber-TRAST, described below) were done in buffered solutions of 4 µM MB and 1 µM IR700. For corresponding tissue measurements, a PBS solution with 8 µM of MB or 2 µM of IR700 was injected through a cannula into a fresh loin of pork. The optical fiber was then inserted through the same cannula for measurements.

### TRAST spectroscopy/imaging—basic concept

In TRAST measurements fluorophore blinking kinetics are determined by recording the average fluorescence intensity, $<F>$, from an ensemble of fluorophores subject to modulated excitation. With the excitation modulation systematically varied on the time scales of the fluorophore dark-state kinetics, rapid blinking kinetics can be quantified without the need for time-resolved detection^19,20^. This enables wide-field cellular imaging of $\mathrm{\mu s}$ blinking kinetics, using a regular camera and exposure times of seconds.

To calculate the recorded fluorescence intensity in the TRAST experiments, we used photophysical models for MB and IR700, as shown in Figs. 3 and 6A, respectively. For both MB and IR700, the singlet state is the only emissive state, with the other states in the model non-luminescent. For a homogeneous solution sample, and from the rate equations of a MB or IR700 fluorophore subject to a rectangular excitation pulse starting at t = 0 (SI Section S1, Eqs. S1–S10), the fluorescence signal recorded by a point detector, or a pixel element of a camera system, can be described by

2 $$F\left(t\right)=c\cdot {q}_{F}\cdot {q}_{D}\iiint \left(CEF\left(\overline{r }\right)\cdot \frac{{{\sigma }_{exc}\Phi }_{exc}\left(\overline{r }\right)}{{\sigma }_{exc}{\Phi }_{exc}\left(\overline{r }\right)+{k}_{10}}\cdot \left[S\right]\left(\overline{r },t\right)\right) dV$$

Here, $\left[S\right]$ denotes the probability that the fluorophore is in a singlet state (either its ground, S_0_, or excited, S_1_, singlet state), ${q}_{D}$ denotes the overall detection quantum yield of the emission from S_1_, ${q}_{F}$ is the fluorescence quantum yield, ${\Phi }_{exc}={{F}_{exc}}^{/\text{hv}}$ is the excitation photon flux, and ${k}_{10}$ the overall decay rate from S_1_. $CEF(\overline{r })$ is the collection efficiency function of the detection system, and $c$ is the fluorophore concentration.

At onset of excitation, $F\left(t\right)$ will show characteristic relaxation on a $\mathrm{\mu s}$ to ms time scale, reflecting changes in the population of the emissive state $\left[S\right]$ (see SI Section S1, Eqs. S1–S10). Similar relaxations can also be observed in the time-averaged fluorescence signal resulting from a rectangular excitation pulse of duration $w$

3 $$\langle {F}_{exc}\left(w\right)\rangle =\frac{1}{w}{\int }_{0}^{w}F\left(t\right) dt$$

when $w$ is increased from the $\mathrm{\mu s}$ to the ms time range. Analyzing so-called TRAST curves, how $\langle {F}_{exc}\left(w\right)\rangle$ varies with $w$, then allows the population kinetics of long-lived photo-induced states of the fluorophore to be determined. This is the general basis for TRAST monitoring^19,20^.

To obtain sufficient photon counts, even for short $w$, we collected the total signal resulting from an excitation pulse train of *N* identical pulse repetitions. *N* is adjusted to maintain a constant laser illumination time, ${t}_{ill}=N\cdot w$, for all $w$. A TRAST curve is then produced by calculating the time-averaged fluorescence signal during excitation for each pulse train, normalized for a given pulse duration, ${w}_{0}$

4 $$\begin{array}{c}{\langle {F}_{exc}\left(w\right)\rangle }_{norm}=\left(\frac{1}{N}\sum_{i=1}^{N}{\langle {F}_{exc}\left(w\right)\rangle }_{i}\right) \Big /\left(\frac{1}{{N}_{0}}\sum_{i=1}^{{N}_{0}}{\langle {F}_{exc}\left({w}_{0}\right)\rangle }_{i}\right)\end{array}$$

The pulse duration used for normalization, ${w}_{0}$, is chosen to be short enough (typically sub-µs) not to lead to any noticeable build-up of dark transient states, yet longer than the anti-bunching rise time of $F\left(t\right)$ upon onset of excitation, which typically is in the nanosecond time range^41^.

In the above expression, ${\langle {F}_{exc}\left(w\right)\rangle }_{i}$ represents the total signal collected from the i:th pulse in the pulse train, as defined in Eq. (3). By using a low excitation duty cycle, here $\eta =0.01$, fluorophores are allowed to fully recover back to S_0_ before the onset of the next pulse, making all pulses in a given pulse train identical. The summations in Eq. (4) are then no longer required and the expression simplifies further. By the normalization step of Eq. (4), several constants cancel out, so that the final expression for ${\langle {F}_{exc}\left(w\right)\rangle }_{norm}$ is independent of $c$, ${q}_{D}$ and ${q}_{F}$. For a uniform excitation photon flux, ${\Phi }_{exc}$, onto the sample, the expression simplifies further, into:

5 $${\langle {F}_{exc}\left(w\right)\rangle }_{norm}=\frac{1}{w}{\int }_{0}^{w}\left[S\right]\left(t\right) dt$$

While a non-uniformity of ${\Phi }_{exc}$ typically has to be accounted for (see TRAST data analysis) this shows the direct connection between $\left[S\right](t)$ and ${\langle {F}_{exc}\left(w\right)\rangle }_{norm}$, as plotted in the TRAST curves. $\left[S\right]\left(t\right)$ can then in turn be given by:

6 $$\left[S\right]\left(t\right)=\sum_{i=1}^{P}\left[{A}_{i}-{A}_{i}{e}^{-{\lambda }_{i}(\overline{r })\tau }\right]$$

Here, ${\lambda }_{i}(\overline{r })$ are the eigenvalues and ${A}_{i}(\overline{r })$ the related amplitudes, reflecting the population build-up of the different photo-induced, non-fluorescent states following onset of a rectangular pulse excitation at $t=0$ (SI Section S1, Eqs. S1–S6).

### TRAST—experimental setup:

TRAST measurements were carried out on a home-built TRAST setup, as previously described^42,43^, based on an inverted epi-fluorescence microscope (Olympus, IX70). Fluorescence was excited by a 638 nm diode laser (Cobolt, 06-MLD, 200 mW) using an excitation filter (Semrock BrightLine 637/7). The laser beam was modulated by an acousto-optic modulator (AOM; AA Opto Electronics, MQ180-A0,25-VIS), defocused by a convex lens, reflected by a dichroic mirror (ZT532/640rpc, Chroma) and then focused close to the back aperture of the objective (Olympus, UPLSAPO 60x/1.20 W) to produce a wide-field illumination in the sample (beam waist *ω*_0_ = 10–25 µm (1/e^2^ radius), depending on alignment). The fluorescence signal was collected by the same objective, passed through the same dichroic mirror, and then passed through an emission filter (ET706/95m, Chroma) before detection by a sCMOS camera (Hamamatsu ORCA-Flash4.0 V3). The experiments were controlled and synchronized by custom software implemented in Matlab. A digital I/O card (PCI-6602, National Instruments) was used to trigger the camera and generate random excitation pulse trains sent to the AOM driver unit. For experiments with modified oxygen concentrations, a stage incubator system (WP and FC-7, Chamlide, Live Cell Instruments) was used. For the fiber-TRAST measurements (Fig. 6), the excitation laser was reflected by a dichroic mirror (ZT640rdc) and coupled into a graded-index multimode optical fiber with a lensed tip (Thorlabs GIF50E, NA 0.2, 50 µm core, 1 m length). The fluorescence generated in the sample at the fiber end was collected and passed through the same fiber, transmitted by the dichroic mirror, and then recorded by the sCMOS camera where an additional emission filter was added (HQ720/150, Chroma) to further reduce background light.

### TRAST data analysis

The TRAST data was analyzed by software implemented in Matlab, as previously described^42,44^. Recorded TRAST data was first pre-processed by subtraction of static ambient background, optional binning to either larger pixels or regions of interest (ROIs) within the recorded images, and correction for bleaching. The bleaching correction was based on 10 reference frames, recorded in between the regular frames throughout the measurements. The overall bleaching was maximally 5–10% of the total detected intensity in the experiments.

A complete TRAST experiment consisted of a stack of 30 fluorescence images. Each image represents the total fluorescence signal from an entire excitation pulse train, captured using a camera exposure time of ${t}_{exp}={t}_{ill}/\eta$, where ${t}_{ill}$ is the total illumination time and $\eta$ is the excitation duty cycle. Pulse durations, $w$, were distributed logarithmically between 100 ns and 1 ms and were measured in a randomized order to avoid bias due to time effects.

In all measurements, TRAST curves were produced by calculating ${\langle {F}_{exc}(w)\rangle }_{norm}$ within a region of interest (ROI) corresponding to a radius smaller than the 1/e^2^ radius of the excitation beam in the sample plane (typically a $15 \mathrm{\mu m}$ radius), and with the ROI centered on this beam. With knowledge of the total laser power onto the sample, the laser excitation intensity distribution was determined from the image of a fluorophore (CF640R succinimidyl ester, product no: SCJ4600044, Sigma-Aldrich) solution, recorded at non-saturating excitation conditions. Each pixel of the reference image was then converted into a local irradiance value. Fitting of photophysical rate parameters was then performed by simulating theoretical TRAST curves using SI Section S1 Eq. S5 based on the photophysical models of MB and IR700 (SI Fig. 6A and SI Section S1, Eqs. S2, S7/S8) and then comparing them to the experimental data. The set of rate parameters best describing the experimental data was determined by non-linear least squares optimization. In the fits, the singlet excited state lifetime, ${\tau }_{f}$, was defined as $1/{\tau }_{f}={k}_{10}+{k}_{ISC}$, where ${k}_{ISC}$ denotes the rate of intersystem crossing, and was for MB fixed to 345ps^28^, and to 4 ns for IR700, as found from time-correlated single photon counting measurements. Excitation cross-sections used in the fits were fixed to calculate ${k}_{01}={{\sigma }_{exc}\Phi }_{exc}$. They were determined by scaling the maximum extinction coefficient given by the supplier of IR700 and from^45^ for MB, with relative difference in the absorption spectrum at 638 nm, which was the wavelength used for excitation. The confidence intervals given for determined rate parameter values of the fitted curves are 95%.

For the fiber-TRAST curves, background measured from corresponding blank samples, and for the different laser powers and pulse durations applied, was subtracted from the measured fluorescence intensities. The beam diameter of the focus produced by the fiber was specified by the manufacturer (Thorlabs) to be 25 µm, with a 30 µm working distance. However, the laser excitation light coming out of the (multimode) fiber likely has a different spatial profile than the excitation beam in the widefield TRAST experiments. Moreover, the fluorescence collected by the same fiber are collected and imaged differently than in the widefield TRAST experiments. After passage through the fiber, the coordinate values of the fluorescence intensity profile recorded by the camera cannot be directly coupled to the corresponding coordinates of the excitation beam in the sample, at the other end of the fiber. To calibrate for these effects, we introduced a scaling factor for the focal beam diameter in the fiber-TRAST experiments, based on comparisons of TRAST curves recorded from the same samples (MB and IR700 in aqueous solution), with widefield- and fiber-TRAST, respectively, and matching the fitting outcomes of these TRAST curves with each other. Based on this calibration, we found that the focal diameter of the excitation laser in the fiber-TRAST measurements should be scaled by a factor of 0.7 giving a diameter of 18 µm (1/e^2^ diameter), when considering a Gaussian beam-profile as was done in the fitting. This scaling factor found to give similar fitted rates for both MB and IR700 measurements, when comparing to the widefield results (SI, Section S3, Fig. S1).

### TCSPC measurements

FCS-curves were recorded and fitted to be used as a comparison to the TRAST-results for IR700 as well as the fluorescence lifetime of this PS. These measurements were done on a TCSPC confocal setup described in SI Sections S4 and S5, together with fitting procedures.

### Supplementary Information

Supplementary Information.

## Data Availability

The experimental raw data behind this study has been placed at the Zenodo repository (10.5281/zenodo.7469900) for open access.

## Abbreviations

AA^−^: Ascorbate
AOM: Acousto-optical modulator
FCS: Fluorescence correlation spectroscopy
FDOT: Fluorescence diffuse optical tomography
FP: Flash photolysis
GSH: Glutathione
IPDT: Interstitial photodynamic therapy
IR700: IRdye700DX
ISC: Intersystem crossing
KI: Potassium iodide
MB: Methylene blue
NIR: Near-infrared
PBS: Phosphate buffered saline
PDT: Photodynamic therapy
PIT: Photo immune therapy
PS: Photosensitizer
ROI: Region of interest
ROS: Reactive oxygen species
TME: Tumor microenvironment
TRAST: Transient state monitoring
