# Supplementary material for: Local monitoring of photosensitizer transient states provides feedback for enhanced efficiency and targeting selectivity in photodynamic therapy
Source: Sci Rep. 2023 Oct 6;13:16829. doi: 10.1038/s41598-023-43625-6 (PMC10558575; doi:10.1038/s41598-023-43625-6)
Supplement: Supplementary file 1 — Supplementary Information. [file 41598_2023_43625_MOESM1_ESM.pdf]

Supplementary part

Local Monitoring of Photosensitizer Transient States Provides Feedback for Enhanced Efficiency and Targeting Selectivity in Photodynamic Therapy.

Elin Sandberg[a], Chinmaya V Srambickal[a], Joachim Piguet[a], Haichun Liu[a], Jerker Widengren[a,*]

[a] Royal Institute of Technology (KTH), Experimental Biomolecular Physics, Dept. Applied Physics, Albanova Univ Center 106 91 Stockholm, Sweden

* Corresponding author: Email: jwideng@kth.se, Phone: +46-8-7907813

S1. Electronic state models

Electronic state models for MB

With the electronic state model shown in Figure 3A, the electronic state population dynamics of a MB fluorophore, subject to a constant excitation photon flux of Φ_{exc} (=$I_{exc}/h\nu$) starting at time t=0, is given by

$$\frac{d}{dt}\bar{A}(t) = M \cdot \bar{A}(t) \qquad \text{(S1)}$$

Here, $\bar{A}(t) = \left[\, [S](t), [T_1](t), [T_1H](t),\ [\dot{R}](t)\right]^T$ represents the population probabilities of the singlet (both S_0 and S_1), triplet, protonated triplet, and photo-reduced (both protonated and non-protonated) state, respectively.

$$M = \begin{bmatrix} -k_{ISC}' & k_{T1} & k_{T2} & k_{OX} \\ k_{ISC}' & -(k_{T1}+k_H+k_{RED}) & -k_{OH} & 0 \\ 0 & k_H & -(k_{T2}+k_{OH}+k_{RED}) & 0 \\ 0 & k_{RED} & k_{RED} & -k_{OX} \end{bmatrix} \qquad \text{(S2)}$$

is the model rate matrix describing the transitions between the states. In the matrix, the effective intersystem crossing rate, from S to T_1 is given by:

$$k_{ISC}' = k_{ISC} \cdot \frac{\sigma_{exc} \cdot \Phi_{exc}}{\sigma_{exc} \cdot \Phi_{exc} + k_{10}} \qquad \text{(S3)}$$

with σ_{exc} denoting the excitation cross section of the singlet ground state, S_0, and k_{10} the decay rate from the excited singlet state, S_1, to S_0. The recovery rate of the photo-radical state, k_{OX}, is a composite recovery rate of the protonated and non-protonated form of the photo-radical. k_{RED} denotes the reduction rate of the T_1 and T_1H states and is for simplicity denoted as one and the same rate from both these states.

The initial condition for Eq. (S1) is

$$\bar{A}(0) = [1, 0, 0, 0\,]^T \qquad \text{(S4)}$$

, assuming all MB fluorophores are in the singlet (ground) state before onset of excitation at $t = 0$.

For a rectangular excitation pulse, Φ_{exc} is constant throughout the excitation duration and the matrix M is not time dependent. The general solution to Eq S1 is then

$$\bar{A}(t) = e^{Mt} \cdot \bar{A}(0) \qquad \text{(S5)}$$

The dependence of the detected fluorescence at time, t, after onset of excitation is then given by

$$F(t) = q_F \cdot q_D \cdot k_{10} \cdot \frac{\sigma_{exc} \cdot \Phi_{exc}}{\sigma_{exc} \cdot \Phi_{exc} + k_{10}} \cdot [S](t) \quad \text{(S6)}$$

For solutions in which the pH is much higher than the pK_A of the MB triplet state (pH >> 7,2) the protonation of T_1 can be neglected. A simplified model (Figure 3C) can then be applied, in which the state population vector of Eq. S1 can then be reduced to $\bar{A}(t) = \left[\ [S](t), [T_1](t), \left[\dot{R}\right](t)\right]^T$, and with the model rate matrix given by

$$M = \begin{bmatrix} -k_{ISC}{}' & k_{T1} & k_{OX} \\ k_{ISC}{}' & -(k_{T1} + k_{RED}) & 0 \\ 0 & k_{RED} & -k_{OX} \end{bmatrix} \quad \text{(S7)}$$

For solutions in which the pH is much lower than the pK_A of the MB triplet state (pH << 7,2), and in which the buffer concentration is high, such that the triplet protonation rate $k_H \gg k_{T1}, k_{RED1}$ and k_{OH}. A simplified model can then be applied, which is analogous to that of Figure 3C, but with $\bar{A}(t) = \left[\ [S](t), [T_1H](t), \left[\dot{R}\right](t)\right]^T$, and with the model rate matrix given by

$$M = \begin{bmatrix} -k_{ISC}{}' & k_{T2} & k_{OX} \\ k_{ISC}{}' & -(k_{T2} + k_{RED}) & 0 \\ 0 & k_{RED} & -k_{OX} \end{bmatrix} \quad \text{(S8)}$$

In Eqs. S7 and S8, k_{OX} refers to the recovery rates of the non-protonated and protonated form of the photo-radical, respectively.

Electronic state model for IR700

With the electronic state model shown in Figure S1,, the electronic state population dynamics of a IR700, subject to a constant excitation photon flux of Φ_{exc} starting at time t=0, is likewise described by Eq. S1, with $\bar{A}(t) = \left[\ [S](t), [T_1](t),\ \left[\dot{R}^-\right](t)\right]^T$ representing the population probabilities of its singlet (both S_0 and S_1), triplet , and photo-reduced state. The model rate matrix is, similar to Eqs. S7 and S8, given by:

$$M = \begin{bmatrix} -k_{ISC}{}' & k_T & k_{OX} \\ k_{ISC}{}' & -(k_T + k_{RED}) & 0 \\ 0 & k_{RED} & -k_{OX} \end{bmatrix} \quad \text{(S9)}$$

In absence of $\dot{R}^-$ formation, only singlet-triplet state transitions have to be considered, with $\bar{A}(t) = [\ [S](t), [T_1](t)]^T$, and

$$M = \begin{bmatrix} -k_{ISC}{}' & k_T \\ k_{ISC}{}' & -k_T \end{bmatrix} \quad \text{(S10)}$$

S2 Triplet state population changes in MB upon addition of KI

With the simplified electronic state model for MB of figure 3C, and with both k_T and k_{ISC} increasing linerly with the concentration of potassium iodide, [KI]:

$$k_{ISC} = k_{ISC}(0) + k_{QISC}[KI] \qquad \text{(S11)}$$

$$k_T = k_T(0) + k_{QT}[KI] \qquad \text{(S12)}$$

, where $k_{ISC}(0)$ and $k_T(0)$ are the ISC and triplet decay rates in the absence of KI, and k_{QISC} and k_{QT} are the linear enhancement factors of these rates upon adding KI.

With $k_{10} \gg k_{ISC}, k_T \gg k_{RED}, k_{OX}$, the steady-state population of T at onset of excitation can be approximated by:

$$\bar{T} = \frac{k_{ISC}'}{k_{ISC}'+k_T} \qquad \text{(S13)}$$

Here, k_{ISC}' is given by $k_{ISC}' = k_{ISC} \cdot \frac{\sigma_{exc} \cdot \Phi_{exc}}{\sigma_{exc} \cdot \Phi_{exc}+k_{10}}$ (Eq. S3), with $k_{01} = \sigma_{exc} \cdot \Phi_{exc}$.

With $k_{10} \gg k_{01}$, and from Eqs. S11-S13:

$$\bar{T} = \frac{\frac{k_{01}}{k_{10}}(k_{ISC}(0)+k_{QISC}[KI])}{\frac{k_{01}}{k_{10}}(k_{ISC}(0)+k_{QISC}[KI])+k_T(0)+k_{QT}[KI]} \qquad \text{(S14)}$$

From Eq. S14, we see that if the relative increase of k_{ISC} is lower than the relative increase of k_T upon adding KI, i.e. if:

$$\frac{k_{QISC}}{k_{ISC}(0)} < \frac{k_{QT}}{k_T(0)} \qquad \text{(S15)}$$

then $\bar{T}$ can be expected to decrease, even if there is a strong external heavy atom effect (EHA) effect and with $k_{QISC} \gg k_{QT}$.

In our TRAST measurments, we determined $k_{ISC}(0)$=1150 μs^{-1} , $k_T(0)$=0.8 μs^{-1} , k_{QISC}=3.4x10^9 $M^{-1}s^{-1}$ and k_{QT}=6x10^6 $M^{-1}s^{-1}$ for MB in an air-saturated PBS buffer (pH 9).

With pH set to 4, we obtained $k_{ISC}(0)$=1150 μs^{-1} , $k_T(0)$=0.3 μs^{-1} , k_{QISC}=4.9x10^{10} $M^{-1}s^{-1}$ and k_{QT}=1.8x10^8 $M^{-1}s^{-1}$.

Thus, in both cases, Eq. S15 is fulfilled, and we see a clear reduction in $\bar{T}$ (Figures 5A and 5B), even with strong EHA effects present.

S3. Fiber-based TRAST measurements

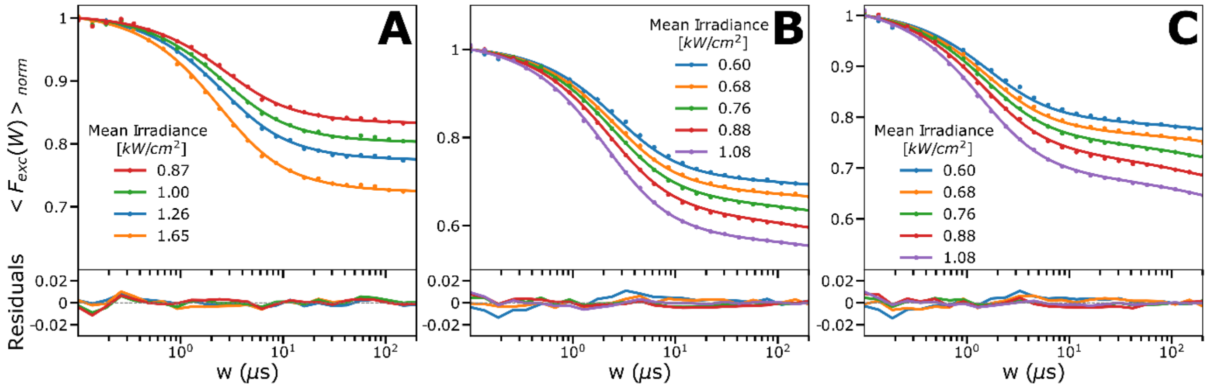

Figure S1. *Experimental TRAST curves, recorded via an optical fiber placed in a PBS (12mM) solution. TRAST data is represented by dots and the lines are the fitted curves with residuals below. More details are given in the main text. Fitted rate parameter values are presented in Table 2. (**A**) I_{exc} dependence of IR700 globally fitted with a triplet state model. (**B**) I_{exc} dependence of MB measured at pH 3.5, where data has been fitted by a 4-state model (**C**) I_{exc} dependence of MB measured at pH 9.3, where again data has been fitted by a 4-state model.*

S4. FCS – experimental setup and analysis

FCS measurements were performed on a commercial, epi-illuminated, confocal laser scanning microscope (Olympus FV1200). Solution samples with IR700 were excited by the focused beam (338nm 1/e² radius) of a 640 nm diode laser (LDH-D-C-640, PicoQuant GmbH, Berlin) in continuous wave. The emitted fluorescence was collected back through the microscope objective (UPlanSApo 60x/1.2w, Olympus), passed through a dichroic mirror (ZT405/488/635rpc-UF2, Chroma), an emission filter (HQ720/15, Chroma), and focused onto a pinhole (50µm) in the back focal plane. The fluorescence signal was finally split and directed on two avalanche photodiodes (Picoquant, Tau-spad), whose signals were collected by a data acquisition card (Hydraharp 400, Picoquant, Berlin).

In the FCS measurements, for freely diffusing fluorescent molecules undergoing dark state transitions, the autocorrelation curves of the recorded fluorescence intensity, $F(t)$, can be described by:

$$G(\tau) = \frac{<F(t)F(t+\tau)>}{<F(t)>^2} = G_D(\tau)G_T(\tau) + 1 \qquad \text{(S16)},$$

where $G_D(\tau)$ denotes the translational diffusion-dependent part $G_T(\tau)$ signifies the contribution from photo-induced dark state transitions. $G_D(\tau)$ can be expressed as:

$$G_D(\tau) = \frac{1}{N_m}\left[1 + \frac{\tau}{\tau_D}\right]^{-1} \times \left[1 + \left(\frac{\omega_0}{\omega_z}\right)^2 \frac{\tau}{\tau_D}\right]^{-\frac{1}{2}} \qquad \text{(S17)},$$

with ω_0 and ω_Z denoting the distances from the center of the laser beam focus in the radial and axial direction respectively at which the collected fluorescence intensity has dropped by a factor of $1/e^2$ compared to its peak value. N_m is the mean number of fluorescent molecules within the detection volume. τ_D is the characteristic diffusion time of the fluorescent molecules, given by the diffusion coefficient D as $\tau_D = \omega_0^2/4D$.

If no dark state transitions occur, the blinking term $G_T(\tau) = 1$. Otherwise, for a fluorophore with *n* dark transient states, and for τ much longer than the anti-bunching relaxation times of the fluorophores, $G_T(\tau)$ can be expressed as a normalized set of relaxation terms [16], averaged over the confocal detection volume, weighted by the square of the detected molecular brightness of the molecules, $W(\bar{r})$:

$$G_T(\tau) = \frac{\int W^2(\bar{r})\left[1 - \sum_{i=1}^{n}\left[A_i(\bar{r}) - A_i(\bar{r})e^{-\lambda_i(\bar{r})\tau}\right]\right]dV}{\int W^2(\bar{r})\left[\sum_{i=1}^{n}\left[1 - A_i(\bar{r})\right]\right]dV} \qquad \text{(S18)}$$

In analogy to Eq. 6, $\lambda_i(\bar{r})$ are the eigenvalues and $A_i(\bar{r})$ the related amplitudes, reflecting the population build-up of the different photo-induced non-fluorescent states. At steady state and with no photobleaching, the sum of the population probabilities for S_0 and S_1, together with $\sum_{i=1}^{n}[A_i(\bar{r})]$ equals one.

S5. Fluorescence lifetime measurements

Time-correlated single photon counting (TCSPC) lifetime measurements were performed on IR700 solutions, prepared as described above, using the same microscope setup as for the FCS measurements, but in this case with the diode laser (638 nm, LDH-D-C-640, PicoQuant GmbH, Berlin) operated in pulsed mode. Instrument response functions (IRFs) were determined from the back-reflected light from the laser excitation pulses. The signals were fed into a data acquisition card (Hydraharp, Picoquant GmbH), deconvoluted and then fit to an exponential decay based on non-linear least squares minimization (Symphotime, Picoquant GmbH).
